# Health Sciences-Evidence Based Practice questionnaire (HS-EBP) for measuring transprofessional evidence-based practice: Creation, development and psychometric validation

**DOI:** 10.1371/journal.pone.0177172

**Published:** 2017-05-09

**Authors:** Juan Carlos Fernández-Domínguez, Joan Ernest de Pedro-Gómez, José Miguel Morales-Asencio, Miquel Bennasar-Veny, Pedro Sastre-Fullana, Albert Sesé-Abad

**Affiliations:** 1 Department of Nursing and Physiotherapy, University of the Balearic Islands, Palma de Mallorca, Balearic Islands, Spain; 2 Department of Nursing, Faculty of Health Sciences, University of Malaga, Andalucía, Spain; 3 Research Group on Evidence, Lifestyles & Health, Research Institute on Health Sciences (IUNICS), Universitat Illes Balears, Palma de Mallorca, Baleares, Spain; 4 Balearic Islands Health Service, Son Llatzer Hospital, Palma, Spain; 5 Department of Psychology, University of the Balearic Islands, Palma de Mallorca, Balearic Islands, Spain; University of Michigan, UNITED STATES

## Abstract

**Introduction:**

Most of the EBP measuring instruments available to date present limitations both in the operationalisation of the construct and also in the rigour of their psychometric development, as revealed in the literature review performed. The aim of this paper is to provide rigorous and adequate reliability and validity evidence of the scores of a new transdisciplinary psychometric tool, the Health Sciences Evidence-Based Practice (HS-EBP), for measuring the construct EBP in Health Sciences professionals.

**Methods:**

A pilot study and a subsequent two-stage validation test sample were conducted to progressively refine the instrument until a reduced 60-item version with a five-factor latent structure. Reliability was analysed through both Cronbach’s alpha coefficient and intraclass correlations (ICC). Latent structure was contrasted using confirmatory factor analysis (CFA) following a model comparison aproach. Evidence of criterion validity of the scores obtained was achieved by considering attitudinal resistance to change, burnout, and quality of professional life as criterion variables; while convergent validity was assessed using the Spanish version of the Evidence-Based Practice Questionnaire (EBPQ-19).

**Results:**

Adequate evidence of both reliability and ICC was obtained for the five dimensions of the questionnaire. According to the CFA model comparison, the best fit corresponded to the five-factor model (RMSEA = 0.049; CI 90% RMSEA = [0.047; 0.050]; CFI = 0.99). Adequate criterion and convergent validity evidence was also provided. Finally, the HS-EBP showed the capability to find differences between EBP training levels as an important evidence of decision validity.

**Conclusions:**

Reliability and validity evidence obtained regarding the HS-EBP confirm the adequate operationalisation of the EBP construct as a process put into practice to respond to every clinical situation arising in the daily practice of professionals in health sciences (transprofessional). The tool could be useful for EBP individual assessment and for evaluating the impact of specific interventions to improve EBP.

## Introduction

Since the middle of the 90s, Evidence-Based Practice (EBP) has become an increasingly important paradigm in health care, as it provides a framework for resolving problems related to everyday clinical practice. EBP assessment in healthcare related professions is usually conducted in the form of self-reported instruments [1–4]. This is due to the impossibility of conducting standardised observation of individual professional practice, from the point of view of both human and material resources.

Most of the EBP measuring instruments available to date present limitations both in the operationalisation of the construct and also in the rigour of their psychometric development, as revealed in the literature review performed [1]. Shortcomings have been detected with respect to their design and development, and the processes of psychometric validation, that is, the provision of solid evidence of reliability and validity. Hence, it still remains to develop tools that rigorously operationalise the EBP construct and submit its items to obtaining adequate evidence of reliabilty and validity [5].

Some systematic reviews have revealed the low prevalence of instruments aimed at measuring EBP from a transdiciplinary perspective [5–9], even though this is considered an important characteristic for their potential usefulness [4]. The first instruments to be developed on EBP from this perspective turned out to be very poor as far as evidence of their psychometric properties were concerned [1,6,7,10]. Neither was their latent structure adequately assessed, and emphasis was placed mostly on the sole identification of barriers and/or facilitators to the use of EBP. Along these lines, the recent proposals of instruments concerning EBP, such as the one by Kaper et al [9], continue to present problems as regards the lack of consideration of the EBP measuring process as a whole, that is, understanding practice as an inherently dynamic process.

Attempts to operationalise the process based on a deeper theoretical analysis of the construct did not include all the steps in said process. Besides, in all cases they were designed for application in a single discipline [1,3,4] and, from the evidence provided, continue to present significant shortcomings in their psychometric behaviour [11–13]. By way of example, in the McEvoy transprofessional instrument [5], which despite being able to be considered one of the most adequate ones to date, the operationalisation of the construct was not comprehensive and its field of validation was reduced to academic competencies. Thereby, the instrument excluded aspects related to the work context or practice setting, resources and support [5].

In order to address the shortcomings and needs pointed out in the literature, the aim of this study was to undergo a psychometric validation of a new transprofessional tool that aims to measure the EBP construct through a latent structure that is able to cover the core contents of the areas of interest included in its theoretical definition.

## Materials and methods

Psychometric validation process was conducted in three stages, following the standards published for the elaboration of psychological and educational tests by the American Psychological Association (APA) and the International Test Commission (ITC) [14–16], as well as the COSMIN protocol for the assessment of the quality of measures in the field of health [17].

Stage 1 covered the HS-EBP development. From the theoretical frameworks more used for EBP and its barriers and facilitators [18–21], a multidisciplinary team composed by experts in EBP developed a first proposal of items to be included. This scheme included five dimensions, with their operational definition (areas of interest): Beliefs-Attitudes (perceived importance, priority, motivation and/or willingness to participate in activities related to EBP, impact, repercussion on patients and relevance), Results of scientific research (posing uncertainty questions to be searched in sources of evidence, appraisal of findings and its application to clinical practice), Development of profesional practice (use of professional experience in problem solving), Assessment of results (knowledge and use of results measures, information analysis and making decisions based on information analysis) and Barriers-Facilitators (contextual and structural support, culture for EBP). This conceptual model is available elsewhere [22]. Finally, a series of items were proposed to be assigned to each of the areas, some of which were created ex novo while others were obtained from the ones included in other existing questionnaires, which had been identified based on a series of systematic reviews of the scientific literature [1,3,4].

Stage 2 consisted of obtaining evidence of apparent and content validity of the HS-EBP questionnaire through two differentiated Delphi studies. The first was conducted with a group of 48 professionals who were recent graduates from four selected key professions: medicine, nursing, physiotherapy and psychology; and in the second case based on a group of 32 experts in EBP from the aforementioned key professions [22].

Finally, stage 3, the aim of the present paper, comprised the process used to assess the rest of the psychometric properties of the HS-EBP questionnaire from a pilot test and a subsequent sample validation test in turn conducted in two phases.

### Participants

Both for the pilot test and for the sample validation test, professionals belonging to Health Sciences were recruited, particularly to Medicine, Nursing, Physiotherapy, and Psychology. The pilot test sample was extracted only from Balearic Islands, and the validation sample from all the Spanish country through a non-probability sampling of volunteers.

### Procedure

Both the pilot test and the sample test were cross-sectional, multicentre, validation studies. All the participants voluntarily completed the corresponding electronic version of the HS-EBP questionnaire implemented through the online survey creation tool “Limesurvey” (https://www.limesurvey.org/es/). A Likert scale ranging from 1 to 10 was used for all items according to the degree of agreement with the statements they contained: the higher the score, the greater the degree of agreement. In all the versions of the questionnaire, additional items were added to collect data related to sociodemographics and practice.

The pilot study was conducted on the 73-item version of the HS-EBP questionnaire resulting from the prior Delphi studies to obtain evidence of apparent and content validity [22]. Meanwhile, the sample validation test was carried out on the 72-item version that arose from the pilot test. After analysing the psychometric properties of the obtained scores, a 60-item reduced version was extracted. The measurement model showed a five-factor structure: Beliefs and attitudes (D1), Results from scientific research (D2), Development of professional practice (D3), Assessment of results (D4) and Barriers/Facilitators (D5). In the sample validation test, the subjects had to complete the rest of the instruments included therein in order to increase the nomological network of the EBP construct and to obtain evidence of criterion validity.

The computerised protocol included the criterion variables Knowledge/Skills and Practice from the Spanish adaptation of the Evidence-Based Practice Questionnaire (EBPQ-19) [23]; the Spanish adaptation of the Scale on Resistance to Change (RTC) [24]; the Spanish version of the Maslach Burnout Inventory (MBI) [25]; and the “Intrinsic Motivation” factor from the Professional Quality of Life questionnaire (CVP-35) [26,27]. All of these showed adequate evidence of reliability and validity in their respective psychometric validation studies. A negative relationship between EBP and RTC was expected, such that individuals who have a greater predisposition to resistance to change are less likely to apply EBP. In particular, this relationship was expected between D1 (Beliefs and attitudes) and all the subscales of the RTC as well as between the dimensions related to the “EBP process” (D2, D3 and D4) and the subscales of “Search for routines”, “Short-term focus” and “Cognitive rigidity”. Likewise, a negative relationship was also expected between EBP and burnout, specifically regarding to the dimensions related to the “EBP process” (D2, D3 and D4). Finally, a positive correlation was hypothesised between EBP and the “Intrinsic motivation” subscale of the CVP variable.

### Data analysis

Data analysis was carried out using SPSS Statistics 20.0 (Chicago, IL, USA) and LISREL 8.8 [28]. Only the results of the subjects who had filled in all the items in the HS-EBP questionnaire were taken into account, such that incomplete protocols were eliminated. No data imputation methods were applied.

In the pilot test, an analysis of internal consistency was performed (Cronbach’s alpha) for the scores of each latent factor in the questionnaire, and then an Exploratory Factor Analysis (EFA) after an initial review of the data to determine their suitability for this analysis [29,30]. A factor extraction method was conducted using Principal Component Analysis (PCA) by applying the Kaiser criterion, and the structure was optimised with a Varimax rotation. These analyses were implemented in order to refine the psychometric behaviour of the items in the version from the prior Delphi studies.

The sample validation test was performed in two stages. In the first stage, the same type of analysis described for the pilot study was conducted in order to obtain a more parsimonious reduced version with a better goodness-of-fit for the psychometric properties of the scores obtained. Thereby, all items that showed worse psychometric behaviour using three qualitative assessment criteria for each individual item were eliminated or reformulated: a) results of the reliability analysis of the dimension upon eliminating each item, b) factor loadings of the items in the EFA, and c) results obtained in the analysis of the content validity evidence of each item (prior Delphi study) regarding its relevance criterion [22].

About the reduced version of the HS-EBP questionnaire, scores’ reliability was analysed through Cronbach’s alpha, and the Intraclass Correlation Coefficient (ICC) for the 5 latent factors [31]. As regards the evidence of validity of the measurement model, a Confirmatory Factor Analysis (CFA) was performed using the maximum likelihood method, after checking the multivariate normality assumption through the PRELIS 2 programme included in LISREL 8.8. Its purpose was to contrast the latent dimension structure a priori in accordance with the operationalised definition of the EBP construct.

To assess the overal fit of the model, the following goodness-of-fit indexes were used: χ^2^, the χ^2^*/df* function, the *Root Mean Square Error of Approximation* (*RMSEA*), its confidence interval at 90%, and the value of *p*(*RMSEA*<0.05), as well as the *Standardized Root Mean Squared Residual* (*SRMR*), the *Comparative Fit Index* (*CFI*) and the *Goodness-of-Fit Index* (*GFI*). A model comparison approach was used considering several latent structures: one-factor, three-factor (by adding the scores related to the “EBP process”, that is dimensions D2, D3 and D4 of the questionnaire) and five-factor model. A Chi-square test on the discrepancy values and the *Akaike Information Criterion* (*AIC*) were obtained to compare the relative fit between models. A model was considered to fit the data if χ^2^ was not significant, χ^2^*/df* <3, *RMSEA*<0.05 or *p(RMSEA<0*.*05)≥0*.*05*, *SRMR*<0.08, and *CFI* ≥0.95 [32,33]. Analytic fit for the factor loadings were also assessed [34] and the correlations between latent factors were also analysed. A 95% confidence level was adopted for the statistical significance of factor loadings.

The evidence of criterion validity of the scores obtained through the non-parametric correlations was assessed, as the normality assumption of the distribution of most of the variables was not fulfilled. Correlations between the dimensions of the HS-EBP questionnaire and the criterion variables considered (that were hypothesised to hold a theoretical relationship with the EBP construct) were estimated. Evidence was obtained of convergent validity of the correlations of the scores of the dimensions of the HS-EBP questionnaire with those of the EBPQ-19 questionnaire.

Finally, in order to obtain evidence of decision validity, the instrument’s classification capacity was assessed, by taking the subjects’ prior training in EBP as a discrimination variable. Respondents were classified in 4 groups: no training in EBP, basic training, intermediate training, and advanced training, and their scores were compared in the different dimensions of the questionnaire through one-way ANOVA. In addition, the robust tests of Brown-Forsythe and Welch were applied in the event of failure of the normality assumption, thereby the degree of convergence between the results was analysed.

### Ethical considerations

The study was approved by the Research Ethics Committee (REC) of the University of the Balearic Islands (registration number 3566). The study was conducted according to the ethical guidelines of the Declaration of Helsinki and the privacy of data was respected (Ley Orgánica 15/1999 on the Protection of Personal Data). Explanatory letters of the study were sent to all participants concerning the computerised protocol, which included all the variables considered, and confidentiality of responses was guaranteed. Completing and sending off the questionnaires was considered consent to participate.

## Results

The pilot test was conducted on a sample of 211 Health Science professionals from Balearic Islands. The median age of the subjects was 38 years, with an interquartile range of 17 years, and 66.4% were women. By profession, there were 38.4% nurses, 30.3% physiotherapists, 10.9% doctors, 9.5% psychologists, and 3.8% from other health professions. A Cronbach’s alpha coefficient of 0.87, 0.94, 0.34, 0.86 and 0.86 was obtained for each of the five dimensions of the questionnaire, that is, respectively, for the factors "Beliefs and attitudes” (D1), “Results from scientific research” (D2), “Development of professional practice” (D3), “Assessment of results” (D4) and “Barriers/Facilitators” (D5). The dataset complied with the eligibility criteria for factor analysis: with an adequate value of 0.87 for the Kaiser-Meyer-Olkin index (KMO), and despite a significant result for Bartlett’s test of sphericity (p<0.001). A PCA was performed by applying the Kaiser criterion and a Varimax rotation, obtaining 17 factors with eigenvalues greater than or equal to 1. This structure was clearly inadequate, wherefore the extraction to 5 factors was subsequently forced, and eigenvalues of 17.94, 5.22, 3.60, 3.27 and 2.69 were obtained for D2, D1, D4, D5 and D3 respectively, which enabled 44.83% of the variance to be explained. Based on an analysis of these results, it was decided to reformulate the wording of the items that scored inversely in all the dimensions, as they had obtained the worst results of internal consistency in their dimension and showed abnormal behaviour in the latent structure. Given the low reliability of D3 (13 items) and the inconsistency in the affiliation of its items to any of the factors in the dimensional structure of the questionnaire, it was decided to apply a PCA exclusively on this dimension so as to analyse the behaviour of the items therein. In the forced extraction to a single factor of D3, only 6 items loaded above 0.40 (explaining 18.19% of the total variance). Based on this result, only the items with the best psychometric behaviour were kept, that is a greater consistency in the factor analysed in the PCA (items 9, 7, 13, 11, 10 and 1 ordered from highest to lowest factorial weight), while reformulating the content of items 9 and 1. It was likewise decided to reformulate items 3, 4 and 5, re-reverse items 2 and 12, and eliminate items 6 and 8, as they presented the worst psychometric behaviour. Three new items were created to attempt to cover the areas of interest that had become under-represented as a result of the modifications or eliminations carried out.

The resulting refined version of the pilot test was the object of analysis of the validation sample test. It was performed on a sample of 869 professionals from different professions related to the Health Sciences throughout the whole Spanish country (see **Table 1**).

**Table 1:** Sociodemographic characteristics of the validation sample.

	Male		Female		Total	
**Age**						
	**(n)**	**%**	**(n)**	**%**	**(n)**	**%**
20–29	60	17.24	111	21.30	171	19.7
30–39	73	20.98	159	30.52	232	26.7
40–49	65	18.68	117	22.46	182	20.9
50–59	108	31.03	115	22.07	223	25.7
60–69	37	10.63	19	3.65	56	6.4
70+	5	1.44	0	0	5	0.6
Total	348	100.00	521	100.00	869	100.00
**Profession**						
	**(n)**	**(%)**	**(n)**	**%**	**(n)**	**%**
Medicine	191	54.88	150	28.79	341	39.2
Nursing	50	14.37	203	38.96	253	29.1
Physiotherapy	69	19.83	97	18.62	166	19.1
Psychology	31	8.91	58	11.13	89	10.2
Others^a^	7	2.01	13	2.49	20	2.3
Total	348	100.00	521	100.00	869	100.00
**Geographic zones**						
					(n)	(%)
Andalusia					127	14.6
Aragon					28	3.2
Catalonia					31	3.6
Castilla-Leon					29	3.3
Valencian Com.					137	15.8
Madrid Com					92	10.6
Balearic Islands					252	29.0
Galicia					22	2.5
Basque Country					86	9.9
Others^b^					65	7.5
Total					869	100.00

^a^ Others includes: Pharmacy, Dentistry, Occupational Therapy, Podology, Speech Therapy, and Nutrition.

^b^ Others includes all the country zones where the number of subjects was < = 20: Asturias, Canary Islands, Cantabria, Castilla-La Mancha, Extremadura, Navarra and Murcia.

Reliability analysis on this version of the questionnaire (72 items) obtained the following values of Cronbach’s alpha for the five dimensions: 0.92, 0.96, 0.87, 0.94 and 0.87 (from D1 to D5, respectively). With regard to the factorial structure, previous statistics were adequate: *KMO* = 0.96, despite the fact that the Bartlett’s sphericity test was statistically significant (p<0.001), and the determinant of the correlation matrix between items had a value very close to 0. The PCA forced to 5 factors obtained eigenvalues of 24.44, 5.24, 4.10, 3.40 and 2.34 for D2, D1, D4, D5 and D3, respectively. This model explained 54.90% of the total variance; namely, 33.95% for the factor “Results from scientific research” (D2), 7.28% for the factor “Beliefs and attitudes” (D1), and 5.70%, 4.74% and 3.25% for each of the other three remaining factors, respectively: “Assessment of Results” (D4), “Barriers-Facilitators” (D5) and “Development of professional practice” (D3). An analysis of the psychometric behaviour of the items, both with respect to reliability and validity, enabled the elimination of two items from D1 (items 10 and 4), another two items from D2 (items 15 and 16) and from D3 (items 2 and 7); as well as three items from D4 (items 15, 1 and 5) and another three from D5 (items 3, 13 and 4).

Results of the psychometric analyses conducted on the reduced version (60 items), obtained from the above refinement process, are pointed out below. High internal consistency was confirmed for the 5 dimensions, with values for Cronbach’s alpha of: 0.93, 0.96, 0.84, 0.94 and 0.91, from D1 to D5, respectively. The ICC values for each of the 5 dimensions were: ICC = 0.53 (CI 95%: 0.5-.55) for D1; ICC = 0.63 (CI 95%: 0.61–0.65) for D2; ICC = 0.35 (CI 95%: 0.32–0.37) for D3; ICC = 0.57 (CI 95%: 0.54–0.60) for D4; and ICC = 0.47 (CI 95%: 0.44–0.49) for D5.

In the CFA, the best fit corresponded to the five-factor model, compared to the single-factor and three-factor models. Difference between models was statistically significant in the Chi-square test, and the significant difference between AIC values with respect to the worse fit of the three-factor model support this result (see **Table 2**). All the goodness-of-fit indexes for the five-factor model were adequate except for the Chi-square value, which was statistically significant: χ^2^ = 4906.46 df = 1700 p<0.01; χ^2^/df = 2.89; ICC = 5370.46; RMSEA = 0.049 CI90% RMSEA = [0.047; 0.050] p(RMSEA<0.05) = 0.89; SRMR = 0.067; CFI = 0.99.

**Table 2:** Results for the fit of Model comparison approach about the latent structure of the reduced version of the HS-EBP questionnaire.

Model	χ^2^	df	Δχ^2^	Δdf	p	AIC
Five-factor	4906.46	1700				5370.46
Three-factor	7853.75	1707	2947.29	7	<0.0001	8303.75
Single-factor	44443.96	1710	36590.21	3	<0.0001	44683.96

χ^2^ **=** chi-square test, df = degrees of freedom, Δχ^2^ **=** chi-square difference, Δdf = degrees of freedom difference, P = p-value, AIC = akaike information criterion

In relation to the five-factor model, factor loading of the items was estimated, where all the saturations were statistically significant with t values greater than 2.00 in absolute value. Factor loadings for each dimension are shown in Table 3 (as a result of CFA, each item is hypothesized to be related to a single dimension and the rest of factor loadings are constrained to a null value).

**Table 3:** Item factor loadings in the five-factor model for the reduced version of the HS-EBP questionnaire.

	D1	D2	D3	D4	D5
**CREAC**^a^					
**Item 1.** Utilizar los resultados de investigación es importante para el desarrollo de mi/nuestra práctica profesional.	.71				
**Item 2.** La práctica basada en la evidencia (PBE) ejerce gran impacto sobre mi labor profesional.	.75				
**Item 3.** La PBE debe jugar un papel positivo en mi práctica profesional.	.84				
**Item 4.** Considero que la PBE mejora la calidad y los resultados de las intervenciones.	.83				
**Item 5.** En el ejercicio profesional, la PBE es una herramienta de ayuda para la toma de decisiones.	.79				
**Item 6.** La PBE implica obtener resultados más eficientes.	.66				
**Item 7.** La PBE ayuda a que atendamos de igual forma y con la misma eficacia a las personas.	.59				
**Item 8.** Considero que los resultados de la investigación tienen importancia para mi práctica diaria.	.77				
**Item 9.** Aplicar la PBE se encuentra entre mis prioridades profesionales.	.82				
**Item 10.** Considero motivante aplicar la PBE.	.81				
**Item 11.** Me interesaría mejorar las competencias necesarias para aplicar la PBE.	.68				
**Item 12.** Estoy dispuesto a cambiar las rutinas de mi práctica cuando éstas se demuestren inadecuadas.	.48				
**RESULT**^b^					
**Item 1.** Resuelvo las dudas o preguntas que surgen de mi práctica mediante la búsqueda de resultados científicos actualizados.		.78			
**Item 2.** Me hago preguntas cuya formulación pueda ser contestadas mediante los resultados de la investigación.		.70			
**Item 3.** Utilizo información proveniente de la investigación científica para responder las preguntas que surgen de mi práctica profesional.		.79			
**Item 4.** Utilizo las principales fuentes de información científica en mi disciplina.		.82			
**Item 5.** Soy capaz de llevar a cabo una búsqueda efectiva de la literatura científica en bases de datos electrónicas.		.77			
**Item 6.** Estoy al día de los resultados de investigación relacionados con mi práctica habitual.		.84			
**Item 7.** Conozco los diferentes diseños de estudios científicos que me permitirán responder a mis dudas o mis preguntas.		.78			
**Item 8.** Suelo utilizar procedimientos de ayuda estandarizados para valorar la calidad de la literatura científica.		.78			
**Item 9.** Suelo valorar la calidad de la metodología utilizada en los estudios de investigación que encuentro.		.76			
**Item 10.** Reconozco las posibles variables extrañas o de confusión y las limitaciones de los estudios seleccionados.		.71			
**Item 11.** Soy capaz de interpretar las implicaciones prácticas de los resultados estadísticos.		.69			
**Item 12.** Valoro la relevancia de los resultados de la investigación sobre las futuras intervenciones.		.71			
**Item 13.** Utilizo investigación actualizada para la toma de decisiones habituales en mi práctica profesional.		.90			
**Item 14.** Utilizo documentación procedente de la literatura científica para orientar mis intervenciones hacia una PBE.		.88			
**PRAC**^c^					
**Item 1.** Incorporo los resultados más actualizados de la investigación científica en la resolución de los problemas de mi práctica profesional.			.96		
**Item 2.** Cuando los resultados de la investigación no concuerdan con mi práctica habitual, la cambio para incorporarlos.			.63		
**Item 3.** Repito las intervenciones que me han dado buenos resultados en situaciones no apoyadas por los resultados de la investigación.			.21		
**Item 4.** En mi práctica diaria utilizo el intercambio de opiniones con otros profesionales.			.41		
**Item 5.** Al abordar situaciones no resueltas por la investigación, pido la opinión a profesionales de reconocido prestigio.			.38		
**Item 6.** Las necesidades y preocupaciones inmediatas de los pacientes y/o sus familiares suponen un elemento importante de mi intervención.			.73		
**Item 7.** Informo a mis pacientes para que puedan considerar las diferentes alternativas de intervención que podemos aplicar.			.68		
**Item 8.** Tengo en cuenta la información proporcionada por mis pacientes sobre su evolución para evaluar mis intervenciones.			.78		
**Item 9.** Integro las preferencias, valores y expectativas del paciente en mis intervenciones.			.71		
**Item 10.** Mis actuaciones profesionales están pactadas en función de las preferencias, valores y expectativas de los pacientes.			.60		
**EVAL**^d^					
**Item 1.** Conozco las medidas objetivas de evaluación de resultados más frecuentemente utilizadas en mi área concreta de práctica.				.72	
**Item 2.** Utilizo medidas estandarizadas, basadas en la evidencia científica, para evaluar los resultados de mis intervenciones.				.78	
**Item 3.** Las medidas de evaluación de resultados que utilizo han sido avaladas por la investigación.				.71	
**Item 4.** Valoro de forma crítica los instrumentos/herramientas disponibles para llevar a cabo el análisis de resultados.				.75	
**Item 5.** Utilizo un procedimiento estandarizado de recogida y almacenamiento de la información de mis pacientes.				.69	
**Item 6.** Registro de forma sistemática los resultados obtenidos de la aplicación de los instrumentos o técnicas de valoración sobre mis pacientes.				.73	
**Item 7.** Registro la información relativa a posibles cambios en la evolución de un caso o durante su intervención.				.61	
**Item 8.** Analizo de forma sistemática y continuada la información recogida sobre las intervenciones con mis pacientes.				.83	
**Item 9.** Evalúo los efectos de mi práctica mediante los registros de resultados.				.83	
**Item 10.** Evalúo los resultados de la aplicación de mis decisiones en términos de su eficiencia.				.77	
**Item 11.** Tengo en cuenta los resultados no esperados tras la evaluación de mi práctica.				.75	
**Item 12.** Cuando los resultados no se ajustan a lo esperado, reviso todo el proceso aplicado para analizar las posibles explicaciones que los justifiquen.				.68	
**BARFAC**^e^					
**Item 1.** Puedo acceder a recursos relacionados con la evidencia científica en mi lugar de trabajo.					.53
**Item 2.** En mi lugar de trabajo existen documentos que orientan las intervenciones hacia una PBE.					.66
**Item 3.** Mantenerse actualizado con los resultados de la investigación es una prioridad en mi lugar de trabajo.					.76
**Item 4.** En mi trabajo existen espacios para compartir y discutir los resultados de la investigación científica con otros compañeros.					.74
**Item 5.** La mayoría de compañeros de profesión con los que me relaciono mantienen una actitud favorable hacia el uso de los resultados de investigación en su práctica.					.65
**Item 6.** Los compañeros de otras profesiones con lo que me relaciono fomentan la utilización de los resultados de la investigación en la práctica.					.63
**Item 7.** Mis pacientes exigen que sus tratamientos estén basados en la evidencia científica.					.59
**Item 8.** Mis responsables jerárquicos fomentan la PBE, o si ejerzo exclusivamente de forma autónoma, yo mismo fomento la PBE.					.78
**Item 9.** Las recomendaciones o exigencias existentes en mi entorno de trabajo para el uso de la PBE son suficientes.					.86
**Item 10.** La distribución del tiempo de mi jornada laboral facilita la búsqueda y aplicación de la evidencia científica.					.65
**Item 11.** En mi lugar de trabajo se incentiva/recompensa aplicar una PBE.					.72
**Item 12.** En mi lugar de trabajo es sencillo cambiar patrones de práctica habituales establecidos.					.54

*Note*: The English translation of the reduced version of the HS-EBP questionnaire can be found in S2 File.

^a^ CREAC: Represents D1 (Beliefs-Attitudes).

^b^ RESULT: Represents D2 (Results of scientific research)

^c^ PRAC: Represents D3 (Development of profesional practice).

^d^ EVAL: Represents D4 (Assessment of results)

^e^ BARFAC: Represents D5 (Barriers-Facilitators)

In general, all items obtained moderate factor loadings for the five-factor model, always above .40, ranging from .48 to .84 in D1, from .69 to .90 in D2, from .61 to .83 in D4, and from .53 to .86. Regarding to Dimension 3, 8 from its 10 items obtained adequate loadings (ranging from .41 to .96), and 2 items showed inadequate values: item 4 (.21) and item 6 (.38).

A moderate correlation between all the dimensions of the questionnaire was also obtained, with the highest value in the dimensions related to the “EBP process” (see **Table 4**).

**Table 4:** Correlation matrix between latent factors in the reduced version of the HS-EBP questionnaire.

	D1	D2	D3	D4	D5
**Beliefs and attitudes** **(D1)**	1.00				
**Results of research** **(D2)**	.53**	1.00			
**Development of professional practice (D3)**	.47**	.72**	1.00		
**Assessment of results** **(D4)**	.41**	.62**	.60**	1.0	
**Barriers/Facilitators** **(D5)**	.34**	.60**	.45**	.56**	1.00

*p<0.05

**p<0.001

With respect to evidence of criterion validity, statistically significant negative correlations were found between D2, D3 and D4 (“EBP process”) and D1 (Beliefs and attitudes) and the criterion variables “Search for routines”, “Short-term focus” and “Emotional reaction to imposed change” for the RTC scale, as well as between D5 (Barriers-Facilitators) and “Search for routines” and “Emotional reaction”. Significant negative correlations were also found between these dimensions in the “EBP process” and D5 (Barriers-Facilitators) with the different criterion variables in the MBI scale. Likewise, significant positive correlations were also obtained between all the dimensions in the HS-EBP questionnaire and the subscale of “Intrinsic Motivation” in the CVP-35 scale. Lastly, the existence of positive significant correlations can be appreciated between the dimensions of “Knowledge/skills” and “Practice” for the EBPQ-19 questionnaire and all the dimensions of the HS-EBP questionnaire, which provides evidence of convergent validity for the HS-EBP questionnaire (see **Table 5**).

**Table 5:** Non-parametric correlation matrix between HS-EBP factors and RTC, MBI, CVP-35 and EBPQ-19 subscales.

	Resistance to change (RTC)					Maslach Burnout Inventory (MBI)			Quality of professional life (CVP-35)		EBPQ-19	
	Search for routines	Emot. reaction	Short term focus	Cognit. rigidity	Overall RTC	Emot. exhaus.	Deperson- alisation	Personal fulfil.	Intrinsic motivat.	CVP9 item	Knowl./ Skills	Practice
**Beliefs- Attitudes (D1)**	-.29**	-.21**	-.35**	-.20	-.31**	-.13	-.80	.13	.34**	.22**	.28**	.19**
**Results from scientific research (D2)**	-.31**	-.29**	-.25**	.15*	-.25**	-.19**	-.18**	.26**	.36**	.28**	.53**	.60**
**Professional practice development (D3)**	-.35**	-.31**	-.33**	-.10	-.36**	-.35**	-.45**	.36**	.48**	.39**	.64**	.67**
**Assessment of results (D4)**	-.30**	-.19**	-.23**	.50	-.22**	-.24**	-.22**	.24**	.33**	.23**	.40**	.42**
**Barriers/** **Facilitators (D5)**	-.17*	-.14*	-.10	.80	-.11	-.3**	-.17*	.15*	.25**	.35**	.45**	.41**

* p<0.05

** p<0.001

Finally, in relation to evidence of decision validity, ANOVA results show significant differences between levels of training in all the dimensions of the HS-EBP questionnaire; specifically in D1 (F_3,865_ = 10.58, p<0.0001), D2 (F_3,865_ = 37.25, p<0.0001), D3 (F_3,865_ = 3.57, p = 0.014), D4 (F_3,865_ = 4.56, p = 0.004), and in D5 (F_3,865_ = 6.50, p<0.0001). The robust tests (Welch and Brown Forsythe tests) also obtained statistically significant values for all factors. Post hoc analyses were applied to compare the different pairs of means corresponding to the different levels of training in each of the dimensions. Significant differences were found between the “advanced” level of training and the rest of the training levels in D2. In the other two dimensions related to the process, namely D3 and D4, there were only significant differences between the “advanced” level and the “with no EBP training” level. In relation to the other two dimensions of the HS-EBP questionnaire (reduced version), the most noteworthy was again the existence of significant differences between the “advanced” level and the “with no EBP training” level, in D1 and D5 (see **Table 6**).

**Table 6:** One-way ANOVA for the five factors of HS-EBP questionnaire and the four levels of training in EBP.

	Level of training in EBP			
	No training in EBP Mean (*SD*)	Basic^a^ Mean (*SD*)	Intermediate^b^ Mean (*SD*)	Advanced^c^ Mean (*SD*)
**Beliefs–Attitudes** **(D1)**	97.39 (15.77)^AB^	101.20 (14.63)	101.80 (11.34)^A^	103.74 (12.20)^B^
**Results from scientific research (D2)**	91.48 (24.80)^AB^	91.32 (24.08)^C^	97.09 (20.93)^AD^	111.00 (18.09)^BCD^
**Professional practice development (D3)**	77.09 (11.78)^A^	76.72 (12.98)	78.03 (10.04)	80.10 (11.36)^A^
**Assessment of results** **(D4)**	83.79 (20.21)^A^	85.40 (22.14)	85.66 (19.60)	90.16 (19.58)^A^
**Barriers/** **Facilitators (D5)**	63.72 (22.90)^A^	66.35 (22.98)	64.19 (19.90)^B^	71.80 (23.27)^AB^

*Note*: Within the same dimension, the levels of training in EBP with the same superscript (i.e. “A”, “B”, “C” and/or “D”) are significantly different from a statistical point of view. In all cases the difference is significant with p<0.05.

^**a**^ Basic training: understood as having done an/some introductory course/s to EBP, bibliographic search in electronic databases or similar.

^b^ Intermediate training: understood as, in addition to the above, also having done a/some introductory course/s to research methodology: asking a research quesiton, critical reading of scientific articles, interpretation of statistical results, or similar.

^**c**^ Advanced training: understood as, in addition to the above, also having done a/some training course/s on research: statistics and handling computer programmes e.g.: SPSS, R, Stata; writing scientific articles, or similar.

## Discussion

The aim of this study was to undergo a psychometric validation of a new transprofessional tool to measure the core contents of EBP. The development and psychometric validation process of the HS-EBP questionnaire involved over 1080 professionals from 4 Health Science professions: medicine, nursing, physiotherapy, and psychology. The HS-EBP questionnaire aimed to cover the shortcomings pointed out in accordance with the established methodological design, following the standards recommended by the APA and the ITC for the construction of tests [14–16], and the COSMIN protocol for assessing quality of measures in the field of health [17].

The pilot study and the subsequent sample validation test made it possible to analyse and refine the version of the HS-EBP questionnaire from the prior version obtained from the content validitation process [22], obtaining a reduced version. This reduced version obtained an adequate degree of internal consistency for the five dimensions. As a novel contribution in relation to the EBP measuring instruments published to date, the dimensions of the HS-EBP were subjected to estimation of the ICC, introducing a greater degree of exigency in the estimation of the instrument’s reliability. The results point towards a moderate degree of agreement in the ICC of three of the five dimensions, substantial in D2, and fair in D3, according to the classification of Streiner & Norman [31].

Regarding the latent structure, confirmatory analyses revealed a better fit for the five-factor model, and provide evidence to corroborate the hypothesised dimensional structure. Few instruments concerning EBP have used confirmatory models [10,23]. Thus, from the point of view of the psychometric evidence, confirmatory analysis constitutes one of the strengths of the HS-EBP questionnaire with respect to most of the ones developed to date.

Based on the results of the measuring model and reliability estimation, D3 could be psychometrically improved. This dimension had also presented certain difficulties during the studies conducted to obtain evidence of content validity [22]. Also items 4 and 6 obtained factor loadings lower than .40 and a psychometric refinement is needed, taking into account the operationalised contents. Nevertheless, according to their content validity, they were conserved in this dimension while further studies are carried out. These issues with this attribute are not new in the literature, and they might reflect the difficulty associated with the operationalisation of what is probably the most complex part of assessing the EBP process, due to its complex dynamic nature [19,21,35]. In fact, no previous psychometric instrument in the literature had considered measuring this part of the process. Given the difficulties presented, this dimension must be followed up and possibly improved in subsequent review processes of the instrument by carrying out new sample tests in order to optimise its quality.

The results obtained with respect to the criterion variables considered point towards those practitioners prone to evidence-based being “less resistant” to any situation of change, tending to experience a lower degree of discomfort, lack of enthusiasm, and anxiety when facing situations of profesional change. Moreover, they also showed less concern for change, and more receptivity towards the potential benefits of EBP. Finally, these practitioners were less likely to be oriented towards highly predictable and conventional tasks, procedures or professional surroundings. In addition, this profile of individuals would also show a lower degree of burnout, with fewer feelings of emotional and affective exhaustion, negative attitudes, and/or depersonalisation, and a greater perception of personal fulfilment with their work and intrinsic motivation.

These results may contribute to expand the nomological network and theoretical framework of the EBP construct, but always with the caution of the limitations of a cross-sectional design. However, it is the first time a trans-professional instrument has been developed in which evidence of criterion reliability is obtained with respect to external variables, constituting one of the strengths of this study. For instance, McEvoy’s [5] trans-professional instrument is one of the most complete instruments in terms of its domains structure. Nevertheless, it must be taken into account that it includes only the measurement of the use of EBP, excluding the dimension related to work context or practice environment, due to the fact that the authors developed it initially in the academic field in order to assess the development of competencies in EBP. Kaper´s [9] instrument is another recently developed transdisciplinary instrument; however it is limited to the mere identification of barriers and/or facilitators for the transfer of the results of scientific research into practice, which although important, constitutes only one part of the EBP construct. This same limitation in measuring EBP is common in the pioneer EBP measuring instruments of a trans-disciplinary nature [6–8]. In short, none of these instruments were created based on a comprehensive development process of the operational definition of the EBP construct intended to be measured, as suggested by the standards recommended by the ITC and the APA for the construction of tests [14–16].

From a non-causal but correlational approach, the HS-EBP questionnaire’s scores allowed to differentiate between the “advanced” level of training in EBP and the rest of the levels analysed. It was dimension 2 “Knowledge/skills and behaviours of professionals with respect to the use of results from scientific research” that enabled a better discriminative capacity. However, no difference was produced between the “no training” level and the “basic” level of training in EBP in the scores of any of the dimensions. These results are similar to those obtained by McEvoy et al [5], where no statistically significant differences were found between the different levels of training with respect to the overall attitudes of professionales to EBP. Yet the present study also provides evidence that the “advanced” level obtains significantly higher scores in D1 (Beliefs and attitudes) of the HS-EBP compared to the other levels. This result adds value to the importance that the development of competency in EBP could be deep enough (advanced level). This fact is reinforced by evidence that other studies provide regarding the fact that “attitudes significantly moderate behaviour” [36]. Thereby, the “advanced” level brings about significant changes, not only in the acquisition of knowledge/skills in EBP, but also in positive attitudes and beliefs, and this is reflected in professional practice that is more consistent with the principles of EBP.

By way of limitations of the study, the type of sampling–non random–and the potential bias of self-selection, owing to the voluntary nature of the participation of the subjects, could be identified. However, the undesired effect of these biases may be alleviated by both the size of the sample used and the fact that four professions–well differentiated in their characteristics, which could be considered as representative of the rest of Health-related professions–were represented.

To complete the psychometric validation process, there still remains for the very near future the need to check that the scores from the HS-EBP questionnaire, especially in the dimensions related to the “EBP process”, are able to predict the results of an objective measuring test of EBP obtained through direct observation of the regular daily practice of the professionals. This criterion could be considered a gold standard for obtaining evidence of decision validity.

To counter the social desirability bias inherent in this type of instruments, it is also necessary to consider the objective measurement of knowledge/skills for EBP, as suggested in the conclusions of the most recent systematic reviews regarding EBP measuring instruments in different disciplines related to the Health Sciences, in which the need for the development and assessment of evaluation tools based on objective competency is manifested [3,4]. No less important is the need to obtain evidence concerning the instrument’s sensitivity to change.

### Conclusions

The HS-EBP questionnaire was rigorously developed and the methodological design used made it possible to obtain suitable evidence of reliability and validity regarding its scores through a range of different professions in the field of health sciences. The tool makes it possible to assess the different dimensions of the EBP construct as a process put into practice to respond to every clinical situation (problem) arising in the daily practice of professionals. Thus, it enables all the elements included in the theoretical definition and proposal of operationalisation thereof to be measured.

This includes the assessment of the different components that are started in the clinical reasoning process prior to decision-making: results from scientific research, clinical experience, and the professional’s ability for clinical judgement. It also includes other sources of information that may become part of a professional’s reasoning process, such as those related to the opinions of work colleagues, etc. Finally, it also enables the assessment of results on health as a final component of the process to be evaluated. Likewise, the HS-EBP allows to assess the main factors at individual and organisational level that influence above all this process of clinical reasoning and decision making, such as the very beliefs and attitudes of professionals towards EBP, and the organisational aspects of the healthcare system in which the professionals carry out their practice.

In short, the validity findings of the questionnaire are promising in terms of the use proposed for it in assessing the EBP construct at individual level, and for evaluating the impact of specific interventions to improve EBP. Thus, the HS-EBP questionnaire will enable its use in clinical practice for diagnostic and interventional approaches, and it is recommended to researchers in the field going beyond along this line, so that in future studies thereon and/or their measuring instruments, these criterion variables may continue to be used in order to obtain scientific evidence regarding these aspects. Obtaining all this evidence of validity from different sources contributes to the achievement of an adequate degree of construct validity of the test scores, as an overall unitary concept of validity.

## Supporting information

S1 File Original language (Spanish) of Health Sciences Evidence Based Questionnaire (HS-EBP). (DOCX)

S2 File English version of Health Sciences Evidence Based Questionnaire (HS-EBP). (DOCX)

S3 File Dataset. Pilot study matrix.
(SAV)

S4 File Dataset. Validation study matrix.
(SAV)
