# Supplementary material for: Health Sciences-Evidence Based Practice questionnaire (HS-EBP) for measuring transprofessional evidence-based practice: Creation, development and psychometric validation
Source: PLoS One. 2017 May 9;12(5):e0177172. doi: 10.1371/journal.pone.0177172 (PMC5423642; doi:10.1371/journal.pone.0177172)
Supplement: S1 File — (DOCX) [file pone.0177172.s001.docx]

**
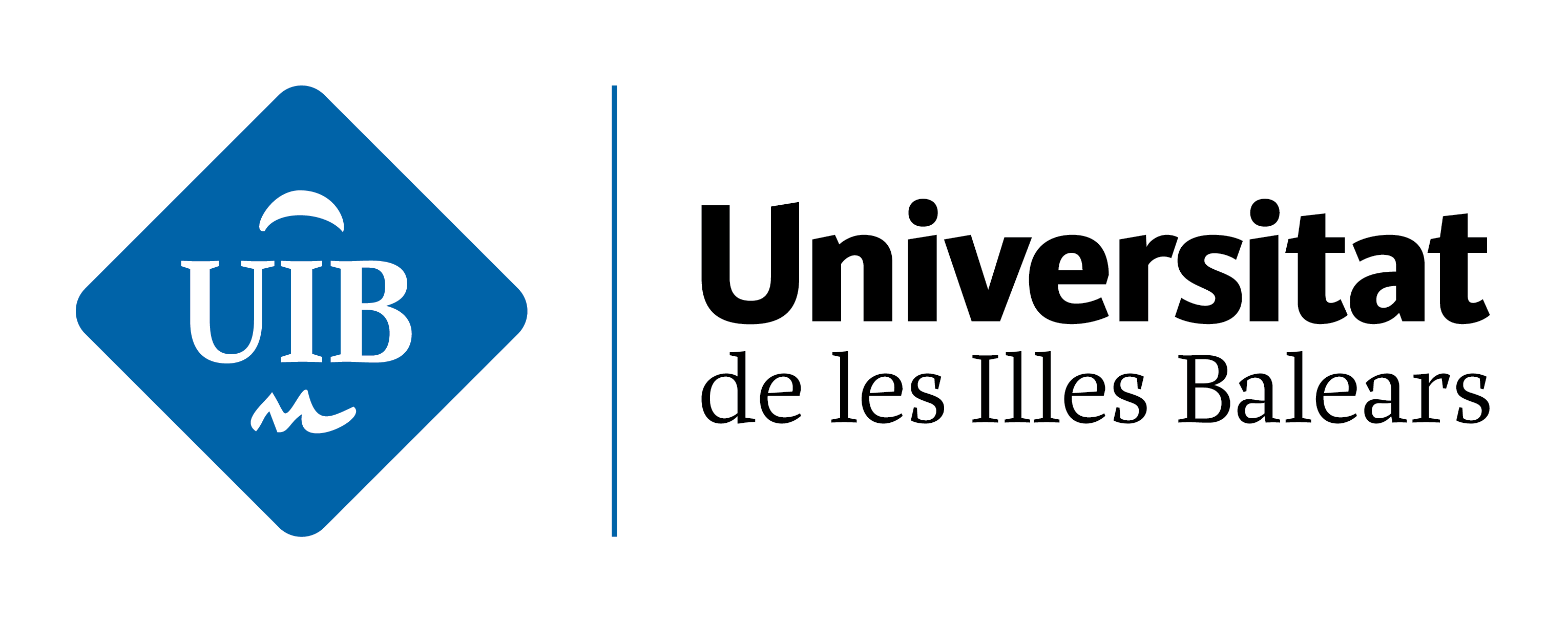
**

**Cuestionario de Práctica Basada en la Evidencia en Ciencias de la Salud (HS-EBP)**

El cuestionario que usted está a punto de contestar está diseñado para recoger información sobre el uso de la Práctica Basada en la Evidencia en Ciencias de la Salud en España.

**CREENCIAS-ACTITUDES**

Esta parte del cuestionario trata de recoger su OPINIÓN acerca de diferentes aspectos relacionados con el paradigma de la Práctica Basada en la Evidencia.

Puntúe en una escala de 1 a 10 su grado de acuerdo con las siguientes afirmaciones (correspondiendo 1 al mínimo y 10 al máximo).

|  | **1** | **2** | **3** | **4** | **5** | **6** | **7** | **8** | **9** | **10** |
| --- | --- | --- | --- | --- | --- | --- | --- | --- | --- | --- |
| 1. Utilizar los resultados de investigación es importante para el desarrollo de mi/nuestra práctica profesional. |  |  |  |  |  |  |  |  |  |  |
| 1. La práctica basada en la evidencia (PBE) ejerce gran impacto sobre mi labor profesional. |  |  |  |  |  |  |  |  |  |  |
| 1. La PBE debe jugar un papel positivo en mi práctica profesional. |  |  |  |  |  |  |  |  |  |  |
| 1. Considero que la PBE mejora la calidad y los resultados de las intervenciones. |  |  |  |  |  |  |  |  |  |  |
| 1. En el ejercicio profesional, la PBE es una herramienta de ayuda para la toma de decisiones. |  |  |  |  |  |  |  |  |  |  |
| 1. La PBE implica obtener resultados más eficientes. |  |  |  |  |  |  |  |  |  |  |
| 1. La PBE ayuda a que atendamos de igual forma y con la misma eficacia a las personas. |  |  |  |  |  |  |  |  |  |  |
| 1. Considero que los resultados de la investigación tienen importancia para mi práctica diaria. |  |  |  |  |  |  |  |  |  |  |
| 1. Aplicar la PBE se encuentra entre mis prioridades profesionales. |  |  |  |  |  |  |  |  |  |  |
| 1. Considero motivante aplicar la PBE. |  |  |  |  |  |  |  |  |  |  |
| 1. Me interesaría mejorar las competencias necesarias para aplicar la PBE. |  |  |  |  |  |  |  |  |  |  |
| 1. Estoy dispuesto a cambiar las rutinas de mi práctica cuando éstas se demuestren inadecuadas. |  |  |  |  |  |  |  |  |  |  |

Las siguientes partes del cuestionario están diseñadas para recoger información sobre conocimientos-habilidades y especialmente sobre el uso de la práctica basada en la evidencia entre profesionales de Ciencias de la Salud.

En ella estamos por tanto interesados especialmente en el USO que usted hace de la evidencia científica y de las distintas fuentes de información disponibles en su práctica. Por tanto le rogamos que trate de responder con la mayor sinceridad posible a las diferentes afirmaciones que se le plantean.

Puntúe en una escala de 1 a 10 (correspondiendo 1 al mínimo y 10 al máximo) el nivel de frecuencia con que realiza las siguientes conductas y/o su grado de acuerdo con las siguientes afirmaciones (según proceda)

**RESULTADOS PROVENIENTES DE LA INVESTIGACIÓN CIENTÍFICA**

|  | **1** | **2** | **3** | **4** | **5** | **6** | **7** | **8** | **9** | **10** |
| --- | --- | --- | --- | --- | --- | --- | --- | --- | --- | --- |
| 1. Resuelvo las dudas o preguntas que surgen de mi práctica mediante la búsqueda de resultados científicos actualizados. |  |  |  |  |  |  |  |  |  |  |
| 1. Me hago preguntas cuya formulación pueda ser contestadas mediante los resultados de la investigación. |  |  |  |  |  |  |  |  |  |  |
| 1. Utilizo información proveniente de la investigación científica para responder las preguntas que surgen de mi práctica profesional. |  |  |  |  |  |  |  |  |  |  |
| 1. Utilizo las principales fuentes de información científica en mi disciplina. |  |  |  |  |  |  |  |  |  |  |
| 1. Soy capaz de llevar a cabo una búsqueda efectiva de la literatura científica en bases de datos electrónicas. |  |  |  |  |  |  |  |  |  |  |
| 1. Estoy al día de los resultados de investigación relacionados con mi práctica habitual. |  |  |  |  |  |  |  |  |  |  |
| 1. Conozco los diferentes diseños de estudios científicos que me permitirán responder a mis dudas o mis preguntas. |  |  |  |  |  |  |  |  |  |  |
| 1. Suelo utilizar procedimientos de ayuda estandarizados para valorar la calidad de la literatura científica. |  |  |  |  |  |  |  |  |  |  |
| 1. Suelo valorar la calidad de la metodología utilizada en los estudios de investigación que encuentro. |  |  |  |  |  |  |  |  |  |  |
| 1. Reconozco las posibles variables extrañas o de confusión y las limitaciones de los estudios seleccionados. |  |  |  |  |  |  |  |  |  |  |
| 1. Soy capaz de interpretar las implicaciones prácticas de los resultados estadísticos. |  |  |  |  |  |  |  |  |  |  |
| 1. Valoro la relevancia de los resultados de la investigación sobre las futuras intervenciones. |  |  |  |  |  |  |  |  |  |  |
| 1. Utilizo investigación actualizada para la toma de decisiones habituales en mi práctica profesional. |  |  |  |  |  |  |  |  |  |  |
| 1. Utilizo documentación procedente de la literatura científica para orientar mis intervenciones hacia una PBE. |  |  |  |  |  |  |  |  |  |  |

**DESARROLLO DE LA PRACTICA PROFESIONAL**

|  | **1** | **2** | **3** | **4** | **5** | **6** | **7** | **8** | **9** | **10** |
| --- | --- | --- | --- | --- | --- | --- | --- | --- | --- | --- |
| 1. Incorporo los resultados más actualizados de la investigación científica en la resolución de los problemas de mi práctica profesional. |  |  |  |  |  |  |  |  |  |  |
| 1. Cuando los resultados de la investigación no concuerdan con mi práctica habitual, la cambio para incorporarlos. |  |  |  |  |  |  |  |  |  |  |
| 1. Repito las intervenciones que me han dado buenos resultados en situaciones no apoyadas por los resultados de la investigación. |  |  |  |  |  |  |  |  |  |  |
| 1. En mi práctica diaria utilizo el intercambio de opiniones con otros profesionales. |  |  |  |  |  |  |  |  |  |  |
| 1. Al abordar situaciones no resueltas por la investigación, pido la opinión a profesionales de reconocido prestigio. |  |  |  |  |  |  |  |  |  |  |
| 1. Las necesidades y preocupaciones inmediatas de los pacientes y/o sus familiares suponen un elemento importante de mi intervención. |  |  |  |  |  |  |  |  |  |  |
| 1. Informo a mis pacientes para que puedan considerar las diferentes alternativas de intervención que podemos aplicar. |  |  |  |  |  |  |  |  |  |  |
| 1. Tengo en cuenta la información proporcionada por mis pacientes sobre su evolución para evaluar mis intervenciones. |  |  |  |  |  |  |  |  |  |  |
| 1. Integro las preferencias, valores y expectativas del paciente en mis intervenciones. |  |  |  |  |  |  |  |  |  |  |
| 1. Mis actuaciones profesionales están pactadas en función de las preferencias, valores y expectativas de los pacientes. |  |  |  |  |  |  |  |  |  |  |

**EVALUACIÓN DE RESULTADOS**

|  | **1** | **2** | **3** | **4** | **5** | **6** | **7** | **8** | **9** | **10** |
| --- | --- | --- | --- | --- | --- | --- | --- | --- | --- | --- |
| 1. Conozco las medidas objetivas de evaluación de resultados más frecuentemente utilizadas en mi área concreta de práctica. |  |  |  |  |  |  |  |  |  |  |
| 1. Utilizo medidas estandarizadas, basadas en la evidencia científica, para evaluar los resultados de mis intervenciones. |  |  |  |  |  |  |  |  |  |  |
| 1. Las medidas de evaluación de resultados que utilizo han sido avaladas por la investigación. |  |  |  |  |  |  |  |  |  |  |
| 1. Valoro de forma crítica los instrumentos/herramientas disponibles para llevar a cabo el análisis de resultados. |  |  |  |  |  |  |  |  |  |  |
| 1. Utilizo un procedimiento estandarizado de recogida y almacenamiento de la información de mis pacientes. |  |  |  |  |  |  |  |  |  |  |
| 1. Registro de forma sistemática los resultados obtenidos de la aplicación de los instrumentos o técnicas de valoración sobre mis pacientes. |  |  |  |  |  |  |  |  |  |  |
| 1. Registro la información relativa a posibles cambios en la evolución de un caso o durante su intervención. |  |  |  |  |  |  |  |  |  |  |
| 1. Analizo de forma sistemática y continuada la información recogida sobre las intervenciones con mis pacientes. |  |  |  |  |  |  |  |  |  |  |
| 1. Evalúo los efectos de mi práctica mediante los registros de resultados. |  |  |  |  |  |  |  |  |  |  |
| 1. Evalúo los resultados de la aplicación de mis decisiones en términos de su eficiencia. |  |  |  |  |  |  |  |  |  |  |
| 1. Tengo en cuenta los resultados no esperados tras la evaluación de mi práctica. |  |  |  |  |  |  |  |  |  |  |
| 1. Cuando los resultados no se ajustan a lo esperado, reviso todo el proceso aplicado para analizar las posibles explicaciones que los justifiquen. |  |  |  |  |  |  |  |  |  |  |

**BARRERAS-FACILITADORES**

Esta última parte del cuestionario pretende recabar información sobre todos aquellos aspectos relacionados con su entorno laboral que usted percibe como BARRERAS o FACILITADORES para la adopción de una Práctica Basada en la Evidencia en su práctica diaria.

Puntúe en una escala de 1 a 10 su grado de acuerdo con las siguientes afirmaciones (correspondiendo 1 al mínimo y 10 al máximo).

|  | **1** | **2** | **3** | **4** | **5** | **6** | **7** | **8** | **9** | **10** |
| --- | --- | --- | --- | --- | --- | --- | --- | --- | --- | --- |
| 1. Puedo acceder a recursos relacionados con la evidencia científica en mi lugar de trabajo. |  |  |  |  |  |  |  |  |  |  |
| 1. En mi lugar de trabajo existen documentos que orientan las intervenciones hacia una PBE. |  |  |  |  |  |  |  |  |  |  |
| 1. Mantenerse actualizado con los resultados de la investigación es una prioridad en mi lugar de trabajo. |  |  |  |  |  |  |  |  |  |  |
| 1. En mi trabajo existen espacios para compartir y discutir los resultados de la investigación científica con otros compañeros. |  |  |  |  |  |  |  |  |  |  |
| 1. La mayoría de compañeros de profesión con los que me relaciono mantienen una actitud favorable hacia el uso de los resultados de investigación en su práctica. |  |  |  |  |  |  |  |  |  |  |
| 1. Los compañeros de otras profesiones con lo que me relaciono fomentan la utilización de los resultados de la investigación en la práctica. |  |  |  |  |  |  |  |  |  |  |
| 1. Mis pacientes exigen que sus tratamientos estén basados en la evidencia científica. |  |  |  |  |  |  |  |  |  |  |
| 1. Mis responsables jerárquicos fomentan la PBE, o si ejerzo exclusivamente de forma autónoma, yo mismo fomento la PBE. |  |  |  |  |  |  |  |  |  |  |
| 1. Las recomendaciones o exigencias existentes en mi entorno de trabajo para el uso de la PBE son suficientes. |  |  |  |  |  |  |  |  |  |  |
| 1. La distribución del tiempo de mi jornada laboral facilita la búsqueda y aplicación de la evidencia científica. |  |  |  |  |  |  |  |  |  |  |
| 1. En mi lugar de trabajo se incentiva/recompensa aplicar una PBE. |  |  |  |  |  |  |  |  |  |  |
| 1. En mi lugar de trabajo es sencillo cambiar patrones de práctica habituales establecidos. |  |  |  |  |  |  |  |  |  |  |
