# Supplementary material for: Health Sciences-Evidence Based Practice questionnaire (HS-EBP) for measuring transprofessional evidence-based practice: Creation, development and psychometric validation
Source: PLoS One. 2017 May 9;12(5):e0177172. doi: 10.1371/journal.pone.0177172 (PMC5423642; doi:10.1371/journal.pone.0177172)
Supplement: S2 File — (DOCX) [file pone.0177172.s002.docx]

**
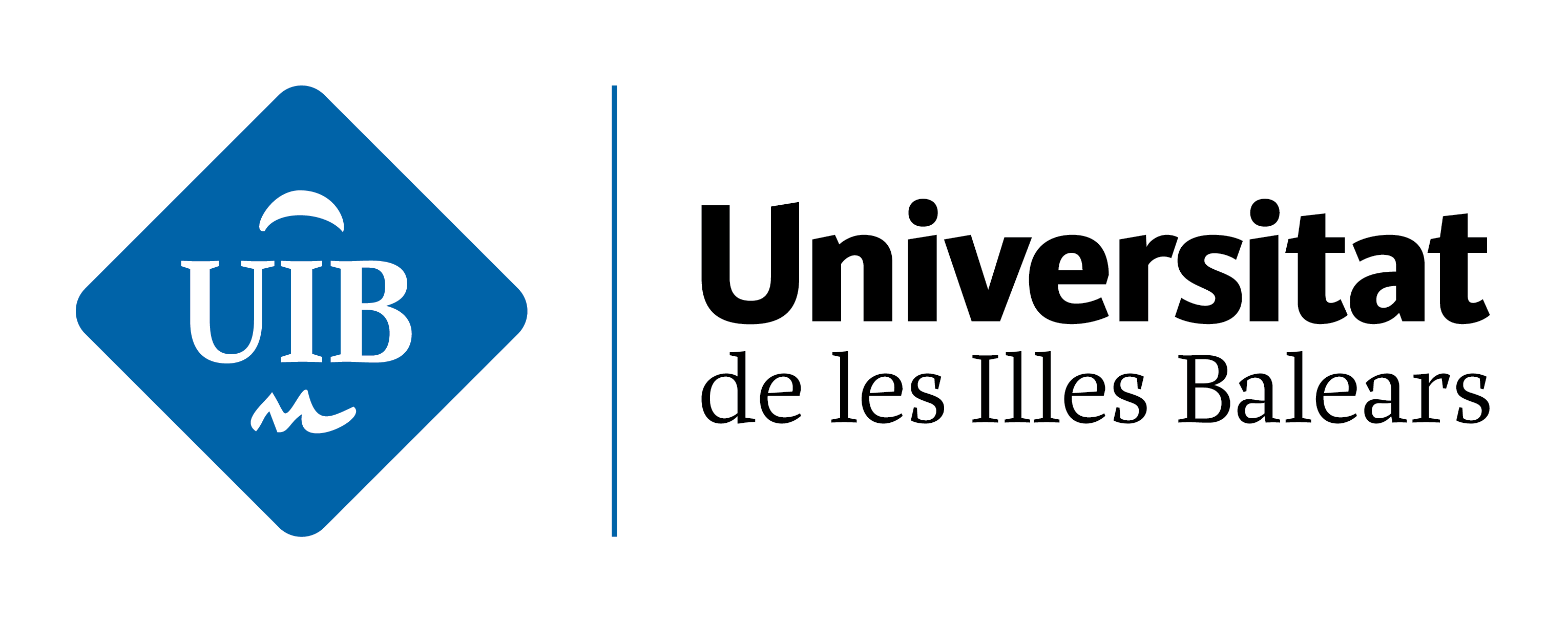
**

**Health Sciences Evidence Based Questionnaire (HS-EBP)**

The questionnaire you are about to answer is designed to collect information on the use of Evidence Based Practice in Health Sciences in Spain.

**BELIEFS-ATTITUDES**

This part of the questionnaire aims to find out your OPINION concerning different aspects related to the paradigm of Evidence Based Practice.

Rate on a scale of 1 to 10 the level of agreement you have with the following statements (where 1 corresponds to the lowest and 10 the highest).

|  | **1** | **2** | **3** | **4** | **5** | **6** | **7** | **8** | **9** | **10** |
| --- | --- | --- | --- | --- | --- | --- | --- | --- | --- | --- |
| 1. Using results from research is important for the development of my/our professional practice. |  |  |  |  |  |  |  |  |  |  |
| 1. EBP has a great impact on my individual practice. |  |  |  |  |  |  |  |  |  |  |
| 1. EBP must play a positive role in my professional practice. |  |  |  |  |  |  |  |  |  |  |
| 1. I consider EBP improves the quality and results of interventions. |  |  |  |  |  |  |  |  |  |  |
| 1. In professional practice, EBP is a helpful tool for decision-making. |  |  |  |  |  |  |  |  |  |  |
| 1. EBP involves getting more efficient results. |  |  |  |  |  |  |  |  |  |  |
| 1. EBP helps us care for people in the same way and with the same efficiency. |  |  |  |  |  |  |  |  |  |  |
| 1. I consider results from research important for my daily practice. |  |  |  |  |  |  |  |  |  |  |
| 1. Applying EBP is among my professional priorities. |  |  |  |  |  |  |  |  |  |  |
| 1. I consider it motivating to apply EBP. |  |  |  |  |  |  |  |  |  |  |
| 1. I am interested in improving the necessary competencies to apply EBP. |  |  |  |  |  |  |  |  |  |  |
| 1. I am willing to change the routines of my practice when these prove inadequate. |  |  |  |  |  |  |  |  |  |  |

The following parts of the questionnaire are designed to gather information regarding knowledge-skills and especially concerning the use of evidence based practice mong Health Science professionals.

In this we are therefore especially interested in the USE you make of scientific evidence and the different sources of information available in your practice. So, we ask you to try to answer the different statements below as sincerely as possible.

Rate on a scale of 1 to 10 (where 1 corresponds to the lowest and 10 to the highest) the degree of frequency with which you carry out the following behaviour and/or the level of agreement you have with the following statements (as appropriate).

**RESULTS FROM SCIENTIFIC RESEARCH**

|  | **1** | **2** | **3** | **4** | **5** | **6** | **7** | **8** | **9** | **10** |
| --- | --- | --- | --- | --- | --- | --- | --- | --- | --- | --- |
| 1. I resolve any doubts or questions arising from my practice by searching for up-to-date scientific results. |  |  |  |  |  |  |  |  |  |  |
| 1. I ask myself questions in such a way that they can be answered through results from research. |  |  |  |  |  |  |  |  |  |  |
| 1. I use information from scientific research to answer questions arising from my professional practice. |  |  |  |  |  |  |  |  |  |  |
| 1. I use the main sources of scientific information in my discipline. |  |  |  |  |  |  |  |  |  |  |
| 1. I am able to carry out an effective search of scientific literature in electronic databases. |  |  |  |  |  |  |  |  |  |  |
| 1. I am up-to-date with the results from research related to my usual practice. |  |  |  |  |  |  |  |  |  |  |
| 1. I know the different designs of scientific studies that will enable me to answer my doubts or my questions. |  |  |  |  |  |  |  |  |  |  |
| 1. I normally use standardised aid procedures to assess the quality of scientific literature. |  |  |  |  |  |  |  |  |  |  |
| 1. I usually assess the quality of the methodology used in the research studies I find. |  |  |  |  |  |  |  |  |  |  |
| 1. I recognize the possible bias or confusion factors and limitations of the studies selected. |  |  |  |  |  |  |  |  |  |  |
| 1. I am capable of interpreting the practical implications of statistical results. |  |  |  |  |  |  |  |  |  |  |
| 1. I assess the relevance of research results on future interventions. |  |  |  |  |  |  |  |  |  |  |
| 1. I use up-to-date research to make habitual decisions in my professional practice. |  |  |  |  |  |  |  |  |  |  |
| 1. I use scientific documentation to guide my interventions towards EBP. |  |  |  |  |  |  |  |  |  |  |

**DEVELOPMENT OF PROFESSIONAL PRACTICE**

|  | **1** | **2** | **3** | **4** | **5** | **6** | **7** | **8** | **9** | **10** |
| --- | --- | --- | --- | --- | --- | --- | --- | --- | --- | --- |
| 1. I incorporate the most up-to-date results from scientific research to solve problems related to my professional practice. |  |  |  |  |  |  |  |  |  |  |
| 1. When results from research do not agree with my usual practice, I change this to incorporate them. |  |  |  |  |  |  |  |  |  |  |
| 1. I repeat interventions that have given me good results in situations not supported by results from research. |  |  |  |  |  |  |  |  |  |  |
| 1. I use exchanges of opinions with other professionals in my daily practice. |  |  |  |  |  |  |  |  |  |  |
| 1. When approaching situations not resolved by research, I ask for the opinion of renowned professionals. |  |  |  |  |  |  |  |  |  |  |
| 1. The immediate needs and concerns of patients and/or their relatives entail an important element of my intervention. |  |  |  |  |  |  |  |  |  |  |
| 1. I inform my patients so they can consider the different intervention alternatives we can apply. |  |  |  |  |  |  |  |  |  |  |
| 1. I take into account information provided by my patients regarding their evolution in order to assess my interventions. |  |  |  |  |  |  |  |  |  |  |
| 1. I integrate the preferences, values and expectations of the patient in my interventions. |  |  |  |  |  |  |  |  |  |  |
| 1. My professional actions are agreed on according to the preferences, values and expectations of patients. |  |  |  |  |  |  |  |  |  |  |

**ASSESSMENT OF RESULTS**

|  | **1** | **2** | **3** | **4** | **5** | **6** | **7** | **8** | **9** | **10** |
| --- | --- | --- | --- | --- | --- | --- | --- | --- | --- | --- |
| 1. I know the objective results assessment measures most frequently used in my specific area of practice. |  |  |  |  |  |  |  |  |  |  |
| 1. I use standardised measures, based on scientific evidence, to assess the results of my interventions. |  |  |  |  |  |  |  |  |  |  |
| 1. The assessment measures I use have been endorsed by scientific evidence. |  |  |  |  |  |  |  |  |  |  |
| 1. I critically appraise the instruments/tools available to carry out the results analysis. |  |  |  |  |  |  |  |  |  |  |
| 1. I use a standardised procedure for collecting and storing information on my patients. |  |  |  |  |  |  |  |  |  |  |
| 1. I systematically record the results obtained from the application of the assessment instruments or techniques on my patients. |  |  |  |  |  |  |  |  |  |  |
| 1. I record information concerning possible changes in the evolution of a case or during the intervention. |  |  |  |  |  |  |  |  |  |  |
| 1. I systematically and continuously analyse the information collected on the interventions with my patients. |  |  |  |  |  |  |  |  |  |  |
| 1. I assess the effects of my practice by recording results. |  |  |  |  |  |  |  |  |  |  |
| 1. I assess the results of applying my decisions in terms of their efficiency. |  |  |  |  |  |  |  |  |  |  |
| 1. I consider unexpected results after assessing my practice. |  |  |  |  |  |  |  |  |  |  |
| 1. When the results do not fit with what is expected, I review the whole process applied in order to analyse possible explanations that may account for them. |  |  |  |  |  |  |  |  |  |  |

**BARRIERS-FACILITATORS**

This last part of the questionnaire aims to collect information on all the aspects related to your work environment that you perceive as BARRIERS or FACILITATORS to adopting Evidence Based Practice in your daily practice.

Rate on a scale of 1 to 10 the level of agreement you have with the following statements (where 1 corresponds to the lowest and 10 to the highest).

|  | **1** | **2** | **3** | **4** | **5** | **6** | **7** | **8** | **9** | **10** |
| --- | --- | --- | --- | --- | --- | --- | --- | --- | --- | --- |
| 1. I can access resources related to scientific evidence in my workplace. |  |  |  |  |  |  |  |  |  |  |
| 1. In my workplace there are documents that guide interventions towards EBP. |  |  |  |  |  |  |  |  |  |  |
| 1. Keeping up-to-date with results from research is a priority in my workplace. |  |  |  |  |  |  |  |  |  |  |
| 1. At work there are spaces to share and discuss scientific research results with other colleagues. |  |  |  |  |  |  |  |  |  |  |
| 1. Most of the colleagues from my profession with whom I relate have a favourable attitude towards using results from research in their practice. |  |  |  |  |  |  |  |  |  |  |
| 1. The colleagues from different professions with whom I relate encourage the use of research in practice. |  |  |  |  |  |  |  |  |  |  |
| 1. My patients demand their treatments be based on scientific evidence. |  |  |  |  |  |  |  |  |  |  |
| 1. My supervisors encourage EBP or, in the event of working independently, I myself encourage EBP. |  |  |  |  |  |  |  |  |  |  |
| 1. There are enough recommendations or demands present in my work environment for the use of EBP. |  |  |  |  |  |  |  |  |  |  |
| 1. Time distribution in my workday facilitates the search for and application of scientific evidence. |  |  |  |  |  |  |  |  |  |  |
| 1. In my workplace the application of EBP is encouraged/rewarded. |  |  |  |  |  |  |  |  |  |  |
| 1. In my workplace changing established patterns of practice is straightforward. |  |  |  |  |  |  |  |  |  |  |

**NOTE:** *This English translation is for guidance purposes only. In its current version it must not be used to measure the EBP construct in English, as this literal translation is pending analysis according to the guidelines of the International Test Commission (ITC) for the adaptation of tests.*
